# Coincident Double Tumor of the Breast and Gastrointestinal Stroma: A Rare Case

**DOI:** 10.7759/cureus.30169

**Published:** 2022-10-11

**Authors:** Kiran Mastud, Yashwant Lamture, Tushar Nagtode, Venkatesh Rewale

**Affiliations:** 1 General Surgery, Jawaharlal Nehru Medical College, Datta Meghe Institute of Medical Sciences, Wardha, IND

**Keywords:** mutation, carcinoma, gastrointestinal, tumor, gist

## Abstract

The most common metastasis sites of malignant stromal tumors are the liver, peritoneum, lung, and bones. Metastasis to the breast is extremely rare. Therefore, this is a very rare combination. Both being primary concomitantly is a further rare combination. Gastrointestinal stromal tumors (GISTs) and other primary carcinomas in the same patient are not unusual, and they are frequently discovered by chance. GISTs are uncommon mesenchymal tumors of the gastrointestinal (GI) system. There is a scarcity of information on the prevalence of mesenchymal tumors and other primary tumors in the literature. Therefore, more studies are required to establish the same. Concomitant GISTs accompany the most prevalent epithelial malignancies of the gastrointestinal system. More research is needed to shed insight into the molecular and genetic pathways of GIST and synchronous tumor oncogenesis and progression. This case report brings to light a synchronous double tumor of the breast and gastrointestinal stroma.

## Introduction

Although it was previously believed that some cases of gastrointestinal stromal tumors (GISTs) are benign (do not spread), it is now understood that all GISTs have some potential to metastasize. Therefore, the origin being benign is contentious. They range in severity from benign, little nodules detected by chance to malignant tumors. They account for less than 1% of all gastrointestinal (GI) tumors. Their clinical presentation varies, which may lead to differential diagnoses requiring careful evaluation. Gastrointestinal stromal tumors (GISTs) start in very early forms of special cells in the wall of the GI tract called the interstitial cells of Canal (ICCs). More than half of GISTs start in the stomach. Most of the others start in the small intestine. A histogenetic derivation from these cells has been proposed. C-kit (receptor tyrosine kinase) (CD117) positivity is found in GISTs. Despite lacking KIT mutations, platelet-derived growth factor receptor (PDGFR) appears to be expressed in some GISTs. The stomach is more likely to develop C-kit or platelet-derived growth factor receptor-alpha (PDGFRA) mutation-driven mesenchymal tumors, which have a variety of specific histologic traits such as spindle cells, epithelial, or pleomorphic shape. The C-kit transmembrane protein, encoded by the kit proto-oncogene (chromosome 4q11-q12), serves as a receptor for stem cell factor (SCF). It has been discovered that C-kit and PDGFRA’s gain of function appears to play a substantial role in the oncogenesis of practically all GISTs [1]. Although GIST has occasionally been connected to other tumors, there is currently no established reason for the association between GIST and breast cancer. Up to 10% of GIST patients had additional malignancies, including clear cell renal carcinoma and cervical, breast, stomach, and lung carcinomas. In this case report, we introduce the rarity of the synchronous occurrence of GIST and breast carcinoma [1].

## Case presentation

Patient information

A 40-year-old housewife came with complaints of a lump in the abdomen for six months. She also complained of loss of appetite, followed by significant weight loss (12 kgs in four months). The patient has no other significant comorbidities. She had regular menstrual cycles. She also underwent an open appendectomy six years back, apart from which the patient has not undergone any other surgeries. There was no history of similar complaints in the patient’s family.

Clinical findings

The general examination went off without a hitch. Per the abdomen examination, a lump of approximately 6 × 6 cm was present in the left hypochondriac region extending up to the left lumbar region, which was firm, mobile, and non-tender. The lump had well-defined margins with no local rise in temperature. Ballotability was present. On further examination, there was a lump of approximately 2 × 2 cm in the outer upper quadrant of the right breast. The lump was firm, non-tender, mobile, and painless with well-defined margins. A simple lymph node of approximately 2 × 1 cm in the posterior group was also palpable. The left breast and left axilla were unremarkable.

Diagnostic assessment

Routine blood investigations were sent (complete blood count, liver function test, kidney function test, random blood sugar, coagulation profile, urine routine, and microscopy) and were within normal limits. Abdominal and pelvic ultrasonography was done and was suggestive of a heterogeneously enhancing hypoechoic mass lesion showing calcific foci and internal vascularity, measuring 9.3 × 9 × 5.9 cm in the left hypochondrium, possibly arising from bowel loops/mesentery.

Ultrasonography of bilateral breasts and axillae was done, and there was evidence of a hypoechoic oval lesion of approximately 19.8 × 12 mm in the outer upper quadrant suggestive of intraparenchymal lymph node, possibly fibroadenoma.

**Figure 1:**
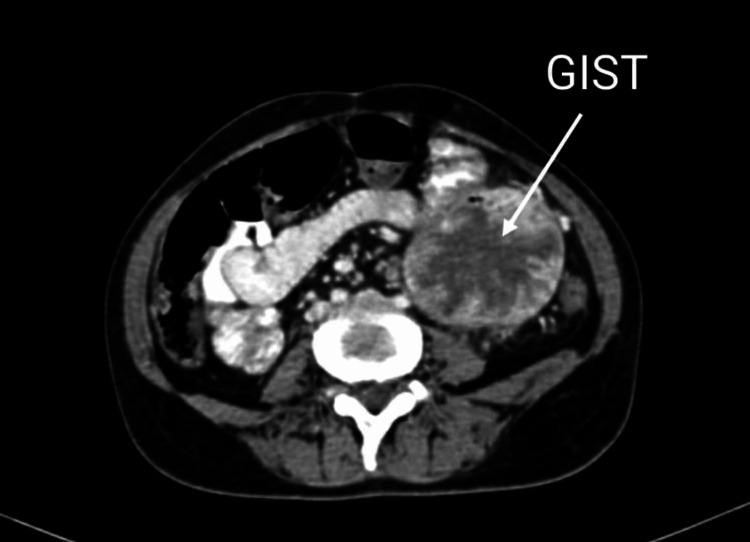
Sagittal view of the contrast-enhanced computed tomography of the abdomen showing a gastrointestinal stromal tumor (GIST) (arrow) arising from the jejunum

Contrast-enhanced computed tomography (CECT) of the abdomen and pelvis were advised and suggested a heterogeneously enhancing large exophytic mass lesion involving the midpart of the jejunum measuring approximately 78 × 65 mm. The lesion showed non-enhancing areas of necrosis with a few calcific foci. The center of the lesion shows a large area of air density, with a possibility of it being an ulcerative lesion (Figure 1).

The lesion was causing a mass effect in the form of displacement of adjacent bowel loops. There was evidence of perilesional fat stranding and multiple subcentimetric to centimetric lymphadenopathy.

There was also evidence of numerous heterogeneously enhancing subcentimetric to centimetric lymph nodes seen in pre-parasitic, aortocaval, central mesenteric, and bilateral paracolic regions, the largest being 1.6 × 1.3 cm in the central mesenteric part. It was associated with the wall thickening of jejunal loops adjacent to the above lesion. Features were suggestive of gastrointestinal stromal tumors arising from the jejunum.

**Figure 2:**
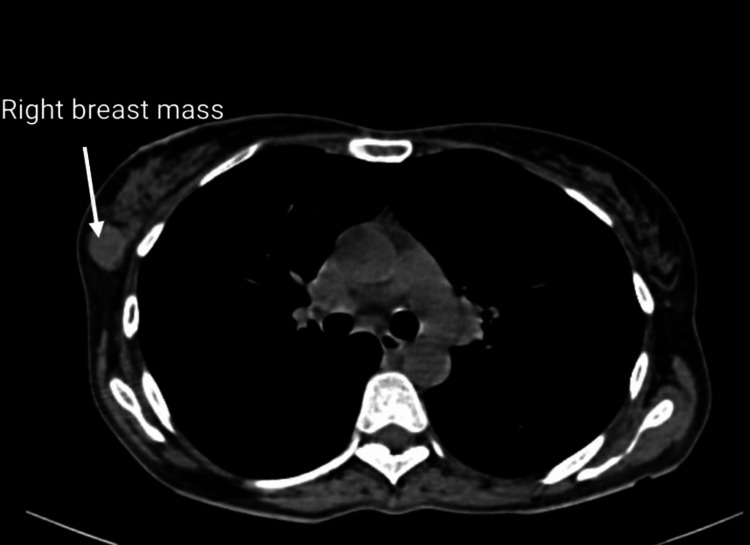
Sagittal view of the computed tomography of the thorax showing a tumor (arrow) in the upper outer quadrant of the right breast

CECT of the thorax was also done and suggested a homogeneously enhancing soft tissue density lesion in the right breast suggestive of fibroadenoma (BIRADS 2), with lymphadenopathy in the bilateral axillary region (Figure 2).

Diagnosis

After undergoing thorough investigations, the patient was diagnosed with a coincident double tumor of the breast and gastrointestinal stroma.

Therapeutic interventions

**Figure 3:**
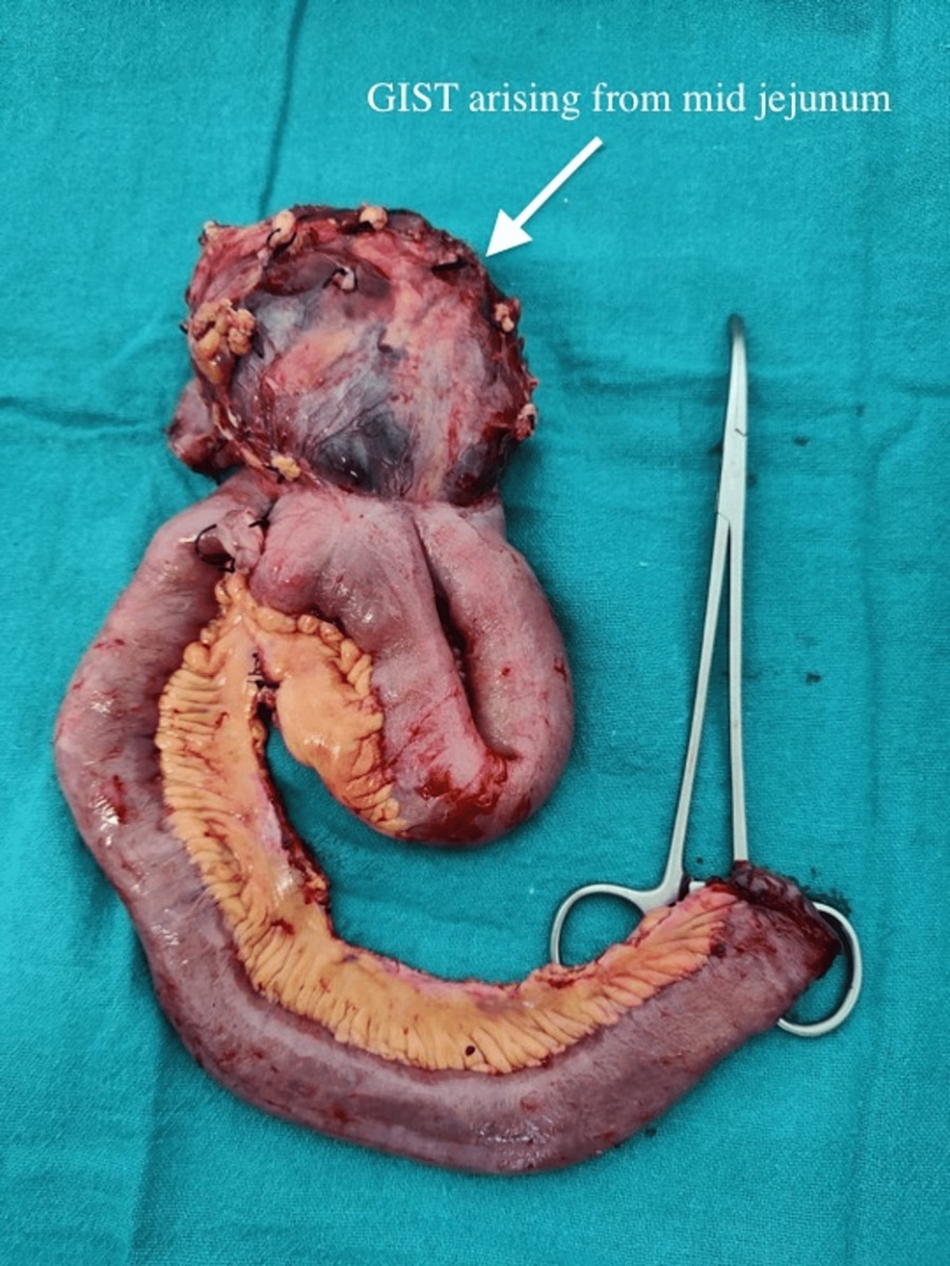
Resected specimen of the gastrointestinal stromal tumor arising from the mid-jejunum

The patient underwent an excisional biopsy of the right breast lump and axillary lymph node followed by exploratory laparotomy for jejunal tumor resection with gastrojejuostomy with jejunojejunal anastomosis done under general anesthesia (Figure 3).

Intraoperatively, an excisional biopsy of the right breast lump and right axillary lymph node was sent for a frozen section, and the report was suggestive of malignant epithelial GIST (metastasis).

However, the histopathology report suggests evidence of metastatic deposits and epithelial malignancy (possibly of breast origin) (estrogen receptor (ER): weak focal positive, progesterone receptor (PR): negative, Her2Neu: positive).

The histopathology report of the jejunal mass was suggestive of gastrointestinal stromal tumor (spindle cell variant). Twelve lymph nodes were identified and were negative for infiltration by malignant cells. Pathological TNM staging (eighth edition) was pT3pNxpMx. In immunohistochemistry studies, the tumor cells expressed C-kit, DOG-1, and SMA and were immune-negative for S-100 protein.

The medical oncologist’s opinion was taken for further management, and tablet imatinib 400 mg once daily (OD) for three years was suggested along with surgery for locally advanced breast cancer (LABC), followed by adjuvant chemotherapy.

## Discussion

The presented case is unusual as the patient was diagnosed with breast carcinoma in an already diagnosed case of gastrointestinal stromal tumor arising from the jejunum. Some experts believe that the association between breast cancer and gastrointestinal stromal tumor (GIST) is just coincidental, while others believe that undiscovered carcinogens cause epithelial and stromal cell proliferation and oncogenesis.

In two significant retrospective studies by Agaimy et al. on the incidence of related non-GIST malignancies in GIST patients, breast cancer had 11% and 7%. The genetic mechanisms of tumorigenesis in the two neoplasms appear to be different, although both GIST and breast cancer have a hereditary predisposition that has been proven. There has been speculation about a potentially shared origin and genetic routes to carcinogenesis, as well as the potential for similar therapeutic approaches, in light of the high occurrence of GISTs in breast cancer patients [2].

Normal breast ductal epithelium and myoepithelial cells have been shown to express C-kit (receptor tyrosine kinase). In contrast, carcinoma cells, which are assumed to be responsible for inhibiting breast cancer cell growth signals, did not.

According to Agaimy et al., 18 (18.6%) of 97 surgically removed GISTs had a subsequent neoplasm (mostly carcinomas), with two (2%) of them having breast malignancies. These two women developed infiltrating ductal mammary cancer, one with two concurrent liver metastases removed in the same setting as the initial GIST and the other with a large malignant GIST (10.5 cm and 12 cm). After 75 and 95 months, respectively, both were still living well. One of them also developed endometrial malignancy later on [2]. In our case, the patient did not have any other metachronous carcinoma but only right breast carcinoma and jejunal GIST. While the therapeutic course in metachronous tumors involves the serial treatment of each tumor, we discovered that personalized and singular treatment in synchronous tumors is assured after an appropriate assessment in multidisciplinary team meetings, and a therapeutic strategy consensus is more effective.

Ludmir et al. described two cases of GIST and primary breast lymphoma (PBL) that occurred simultaneously. Given the dubiousness of the co-occurrence of such rare tumors, there may be a common underlying variation driving both lesions, leading to speculation that these cancers may share common antiapoptotic pathways [3]. However, in our case, the patient was found to have breast carcinoma, not lymphoma. It is probably attributed to the overexpression of C-kit in normal breast ductal epithelium.

In a study piloted on a small sample of patients in 2000, Maiorana et al. provided the first documentation of the occurrence of different cancers in GIST patients, and 52 cases of gastric GIST were examined by the researchers. Six of them had additional tumors, stomach adenocarcinomas in five cases and carcinoid tumors in one case. None of the 52 cases was associated with breast carcinoma, thereby highlighting the rarity of our case [4].

By dividing patients into four groups based on the index tumor and whether the linked neoplasm was synchronous or metachronous, Fernández et al. established a classification based on the temporal connection between GISTs and other malignancies. In 2018, the same authors reported a series of patients with GISTs associated with additional second major tumors, comparing the various subgroups of associated GISTs by the suggested classification. The primary study findings revealed that synchronic diagnoses of GIST and a second primary tumor occurred in 41.2% of patients. The related tumors were discovered before GIST in 23.5% of cases, after GIST in 35.2% of cases, and as synchronous associated neoplasm in 2.9% of cases during GIST staging [5].

## Conclusions

Based on this scenario, we wish to underline the importance of considering the possibility of synchronous and metachronous tumors during the clinical evaluation of patients with malignancies, rather than merely the occurrence of metastatic diseases. In conclusion, primary tumors and GIST might occur together more frequently than previously thought, and they typically become apparent by chance after surgery for another cancer. Even more cases of GIST may be found in patients with adenocarcinoma if the search for them is conducted more aggressively. Hence, initial evaluation is all the more important so as to not miss synchronous tumors. The molecular and genetic processes of carcinogenesis and progression linked to GIST and synchronous tumors need to be clarified through additional research.
